# Validation of the German Glasgow Sensory Questionnaire and replication of sensory processing differences in students with higher and lower Autism-Spectrum Quotient

**DOI:** 10.1186/s12888-023-04903-9

**Published:** 2023-06-14

**Authors:** Annalena Zeisel, Tobias Thiel, Sebastian B. Gaigg, Veit Roessner, Melanie Ring

**Affiliations:** 1 Klinik und Poliklinik für Kinder- und Jugendpsychiatrie und -psychotherapie, Medizinische Fakultät, Technische Universität Dresden, Dresden, Germany; 2 Autism Research Group, Department of Psychology, City, University of London, London, UK

**Keywords:** Autism spectrum disorder, Autism-Spectrum Quotient, Glasgow Sensory Questionnaire, Cross-cultural adaptation, Sensory processing differences

## Abstract

**Background:**

The Glasgow Sensory Questionnaire (GSQ) gives insight into sensory processing differences (hypo- and hyper-sensitivity across modalities), which is a clinically defining characteristic of autism spectrum disorder (ASD). Because there is no validated German version of this instrument, this study aimed at validating the German GSQ. Further, a replication of the GSQ’s sensory processing differences was intended.

**Methods:**

University students of Technische Universität or Universitätsklinikum in Dresden, Germany, were recruited via email distribution or the university homepage and 297 German-speaking students completed the online survey, comprising the German GSQ, Autism-Spectrum Quotient (AQ) and Symptom-Checklist (SCL-90). For validation of the German GSQ, confirmatory factor analyses followed by exploratory factor analyses were applied.

**Results:**

The German GSQ has moderate to low validity, good to acceptable reliability, and a different internal structure from the original GSQ. Replicating the sensory processing differences in students with higher and lower AQ was not successful.

**Conclusions:**

Results indicate that the GSQ, developed especially for individuals with ASD, is less informative for the general population if there are not enough individuals with higher AQ scores in the sample.

## Background

Imagine you are at home: the light in your room has been flickering barely noticeable for weeks, a light breeze comes through the door, and you hear the dull car noises outside. Just another day in an ordinary apartment, for most. What if, for you, the flickering feels like fireworks, the breeze is freezing cold, and the noise upsets you? Every individual perceives identical sensory stimuli differently. Some seek them out specifically, simply accept them, or find them irritating and unpleasant. And for some, such “ordinary” stimuli can become unbearable.

Autism spectrum disorder (ASD) is a neurodevelopmental disorder that is characterized by difficulties in social communication and interaction (A) and restricted, repetitive behavioral patterns (B) [1]. In addition, the fifth version of the Diagnostic and Statistical Manual of Mental Disorders now recognizes that ASD is very commonly associated with sensory processing differences including both hyper- and hyposensitivity [1]. This means that there are individuals with ASD who perceive sensory stimuli differently, either as particularly intense (e.g., background noises seem especially loud and lead to distraction) or much weakened (e.g., hurting oneself but feeling no pain).

A number of studies have documented both sensory hyper- and hyposensitivity in autism using primarily self-report questionnaires [7, 18, 23, 28, 40, 43]. However, qualitative interviews [57, 69] as well was psychophysiological [47] and neuroimaging [12, 25, 26] evidence confirms that individuals with ASD often experience their sensory environment differently. In the general population there is evidence of an association between subclinical autism-like traits as measured with the Autism-Spectrum Quotient (AQ; [6]) and sensory processing differences as captured by the Glasgow Sensory Questionnaire [51]. Sensory processing differences in ASD can affect all sensory modalities, including vision [4, 12, 37, 55], hearing [27, 37, 56, 69], taste [8, 37, 41], olfaction [3, 8, 15, 37], tactile perception [5, 37], vestibular perception [33, 37], and proprioception [37, 47]. Senses can be affected individually, or processing differences can occur in multiple modalities [54].

Sensory processing differences in ASD often go along with sensory seeking (e.g., repetitive sniffing or touching of objects or seeking out visual stimulation) and/or sensory defensive behaviors (e.g., avoiding noisy environments) [14, 40, 43], and are thought to contribute to the development of mental health conditions such as anxiety [2, 21, 22, 31, 46], which commonly co-occur with ASD [30, 34, 65]. Especially individuals with ASD who have difficulty to react adequately to sensory input end up in stress, pain, or anxiety [2, 21, 24, 64]. In fact, 14%-26% of individuals with ASD are affected by a comorbid anxiety disorder [49]. Gillot and Standen [22] report that in adults with ASD elevated anxiety correlated with sensory stress.

Besides anxiety, in adults with ASD, unpleasant sensory sensitivity was related to fear, anger, social difficulties, and escape from certain situations, followed by avoidance, isolation, and depression [57]. Also, Lundqvist [38] found a relation between tactile over-responsiveness and impaired social interaction. Furthermore, hyper- and hyposensitivity led to feelings of sickness, emotional unease, or inability of multisensory processing due to fixation to one sensory input in an adult general population with more autism-like traits [48]. Last, hyper- and hyposensitivity were related to social deficits in individuals with ASD of all ages [62]. However, sensory hypersensitivity was not only related to negative experiences. Adults with ASD reported that heightened sensory experiences also resulted in calming, pleasure, fascination, and possibly went along with newfound abilities and even with professional activities, such as being an excellent chef due to distinct sensitivity regarding the gustatory sense [57]. Whether sensory input is perceived as positive or disturbing depends on the respective situation [57].

To gain insight into sensory processing differences in adults, previous research has used different self-report questionnaires such as the Adolescent/Adult Sensory Profile [9]*,* the Sensory Perception Quotient [60]*,* or the Sensory Sensitivity Questionnaire [44]. The Adolescent/Adult Sensory Profile was not specifically developed for individuals with ASD, and all listed questionnaires cover no more than five different sensory modalities. This has motivated the development of the Glasgow Sensory Questionnaire (GSQ; [48]) as a self-report measure of hyper- and hyposensitivity across all seven sensory modalities for individuals with ASD. It consists of 14 subscales concerning seven sensory modalities (visual, auditory, gustatory, olfactory, tactile, vestibular, proprioception) and the two domains hypersensitivity and hyposensitivity. The GSQ is available in its original language, English [48], and has been validated already in Japanese [59, 63], French [51], Danish [35], and Chinese [67]. It strongly correlates with the AQ [6] in all these translations [31, 35, 48, 51, 59, 63, 66, 67] indicating that, regardless of language, more autism-like traits go along with greater sensory processing differences as indicated by higher scores on the GSQ [31, 48, 51, 63, 67]. In addition, adults with ASD scored higher on the GSQ compared to typically developing adults [35, 59].

Regarding the GSQ, Sapey-Triomphe et al. [51] reported a stronger correlation between the domains hyper- and hyposensitivity, higher hypersensitivity scores in relation to hyposensitivity scores, and a more consistent pattern of sensory processing differences for individuals with higher AQ scores compared to individuals with lower AQ scores. Sapey-Triomphe et al. [51] referred to these results as “sensory profiles”, because the AQ and GSQ scores were related to each other in a particular way, so called “profiles”. This study refers to it as “sensory processing differences”. These findings might implicate that individuals with more autism-like traits experience more sensory processing differences (both hyper- and hyposensitivity) than individuals with fewer autism-like traits. Importantly, the GSQ seems also sensitive to sensory processing differences in other conditions such as attention deficit and hyperactivity disorder, anxiety, history of mental illness, and migraine. Individuals with these conditions also scored higher on the GSQ [31, 45], which is important to bear this in mind when interpreting the GSQ.

As shown above, the GSQ is a helpful tool to capture sensory processing differences. However, to this day there is no German version of the GSQ. Therefore, for the purpose of this study, the English version of the questionnaire was translated, and a large German University student sample was asked to fill in the GSQ as well as the German AQ [20]. The two main goals of this study were: (a) the validation of the German version of the GSQ according to the *Universalist model of cross-cultural adaptation* [29] and (b) the replication of sensory processing differences of individuals with higher or lower AQ scores as reported in the original English version by Robertson and Simmons [48] and in the French version of Sapey-Triomphe et al. [51]. Regarding the first aim, consistent with past research, it was expected that construct validity, reliability, and internal structure of the German GSQ are comparable to the original English version [48]. Regarding the second aim, it was expected that the German student sample would show similar sensory processing differences as the French sample depending on whether participants scored higher or lower on the AQ [51]. More precisely, a stronger correlation between hyper- and hyposensitivity, higher hypersensitivity scores in relation to hyposensitivity scores, and a more homogeneous pattern of sensory processing differences in all seven sensory modalities for individuals with higher compared to individuals with lower AQ scores were expected.

## Methods

### Participants

A sample of 297 German University students was recruited from October 2020 to May 2021 as part of a larger research project with the aim of improving the diagnostic process of anxiety disorders in individuals with ASD. The majority of the sample was recruited via the student email distribution list to 24,408 students including students of the Technische Universität Dresden (TUD) and of the Universitätsklinikum Carl Gustav Carus Dresden (UKD). The remaining participants were recruited via the homepage for online registration for experiments of the Faculty of Psychology of the TUD, the central experimental server of psychology of the TUD, the homepage for students of the UKD, the homepage of the Student Representatives of the UKD, Facebook groups, eBay, word of mouth, and private recruitment of friends and family. Participants were included if they reported being a student of at least 18 years, spoke German fluently, and if they fully completed the questionnaires. Participants were excluded if they reported consuming cannabis more than five times a year or if they used any other illegal drugs within the past year. Inclusion and exclusion criteria as well as demographic data were determined prior participation via a screening questionnaire. Following email and online recruitment, 490 students got in touch. Due to different aspects (minimum of subjects for inclusion of 300, gender ratio, and deadline), only 348 individuals were screened with 310 students fulfilling inclusion criteria. Six of these individuals did not report back, so access to the online survey was sent out to 304 students. Of those, seven individuals did not fill in the survey (reasons e.g., no time, private reason). Therefore, the total sample included 297 German speaking students with varying educational qualifications and fields of study (176 women, 121 men, mean age: 23.5 ± 3.9 years, range: 18–52 years, see Table 1). Because the current study took place during the Covid-19 pandemic, perceived psychological distress was measured using the Global Severity Index (GSI) of the Symptom-Checklist 90-R (SCL-90-R; [16]). Students reported an average GSI T-score of 47.8 (≥ 60 = increased psychological distress). Further, at screening, 16.5% of the students reported having been affected by at least one physical illness (e.g., migraine, thyroid disease, neurodermatitis) and 12.8% by at least one mental illness (e.g., depression, anxiety, eating disorders) in their lives. Detailed information is presented in Tables 1 and 2.

**Table 1:** Demographical data: age, gender, education

	All subjects (*N* = 297)	Students with higher AQ scores (*n* = 35)	Students with lower AQ scores (*n* = 35)
Demographical data	*M (*± *SD)*	*M (*± *SD)*	*M (*± *SD)*
Age	23.5 (± 3.9)	24.4 (± 6.3)	24.1 (± 4.5)
Demographical data	*n (%)*	*n (%)*	*n (%)*
Women/Men	176/121 (59.3/40.7)	19/16 (54.3/45.7)	19/16 (54.3/45.7)
Graduation			
Entry qualification for university of, applied sciences	4 (1.3)	-	-
High school diploma	220 (74.1)	25 (71.4)	29 (82.9)
University of applied sciences	13 (4.4)	3 (8.6)	2 (5.7)
University	60 (20.2)	7 (20.0)	4 (11.4)
Aimed university degree			
Bachelor	111 (37.4)	15 (42.9)	13 (37.1)
Master	60 (20.2)	7 (20.0)	5 (14.3)
Diploma	60 (20.2)	7 (20.0)	12 (34.3)
State examination	64 (21.5)	6 (17.1)	5 (14.3)
Postgraduate	1 (.3)	-	-
Doctoral studies	1 (.3)	-	-
Fields of studies^a^			
Psychology	61 (20.5)	9 (25.7)	6 (17.1)
Mathematics and natural sciences	31 (10.4)	3 (8.6)	2 (5.7)
Civil and environmental engineering	66 (22.2)	11 (31.4)	5 (14.3)
Engineering sciences	33 (11.1)	2 (5.7)	9 (25.7)
Humanities and social sciences	43 (14.5)	5 (14.3)	7 (20.0)
Medicine	32 (10.8)	2 (5.7)	4 (11.4)
Teacher training	31 (10.4)	3 (8.6)	2 (5.7)

^a^Fields of studies were grouped according to the classification of the TUD [61] and an online study guide [10]

**Table 2:** Mental and physical health

	All subjects (*N* = 297)	Students with higher AQ scores (*n* = 35)	Students with lower AQ scores (*n* = 35)
Mental Health	*M (*± *SD)*	*M (*± *SD)*	*M (*± *SD)*
SCL-90-R GSI T-score	47.8 (± 10.6)	54.5 (± 12.2)	52.5 (± 9.3)
Mental and Physical Health	*n (%)*	*n (%)*	*n (%)*
Number nonF disorders			
0	248 (83.5)	29 (82.9)	31 (88.6)
1	41 (13.8)	4 (11.4)	2 (5.7)
2 or more	8 (2.7)	2 (5.7)	2 (5.7)
Number F disorders			
0	259 (87.2)	27 (77.1)	31 (88.6)
1	27 (9.1)	6 (17.1)	3 (8.6)
2 or more	11 (3.7)	2 (5.7)	1 (2.9)
Self-reported F disorders			
F10-19^a^	4 (1.3)	-	1 (2.9)
F30-39^b^	15 (5.1)	3 (8.6)	2 (5.7)
F40-49^c^	12 (4.0)	2 (5.7)	1 (2.9)
F50-59^d^	9 (3.0)	2 (5.7)	-
F60-69^e^	2 (.7)	-	-
F90-99^f^	4 (1.3)	2 (5.7)	1 (2.9)

Psychiatric conditions were grouped to the ICD-10-GM Version 2021 [11]

^a^F10-19—mental and behavioral disorders due to psychoactive substance use

^b^F30-39—mood (affective) disorders

^c^F40-49—anxiety, dissociative, stress-related, somatoform and other nonpsychotic mental disorders

^d^F50-59—behavioral syndromes associated with psychological disturbances and physical factors

^e^F60-69—disorders of adult personality and behavior.

^f^F90-98—behavioral and emotional disorders with onset usually occurring in childhood and adolescence

### Composition of the subgroups for replicating sensory processing differences

To differentiate between individuals with higher or lower AQ scores in this study, a cut-off score of 26 was used, following previous studies [51, 70]. This cut-off value has been suggested to have acceptably high sensitivity and specificity to identify levels of autism-related traits in general population samples that may be clinically relevant (see [50]). In the present study, 262 students had an AQ < 26 points and 35 students an AQ ≥ 26. The 35 students with higher AQ scores were compared with a matched subsample of 35 students with lower AQ scores, derived from the group of 262 students. Individuals with low AQ scores were selected in a way that the two sub-groups of 35 students each did not differ in terms of gender, χ^2^(1) = 0.00, *p* = 1.00, φ = 1.00, and age, *t*(68) = -0.26, *p* = .79, *d* = -0.06, 95% Confidence Interval (CI) [-0.53, 0.41] and were similar in their SCL-90 GSI scores and in the occurrence of any mental disorders. Overall, subgroups did not differ in their demographic data, *U*_min_ = 490.00, *Z* = -1.46, *p*_min_ = .15, *r* = -0.17, their reported mental and physical health, *U*_min_ = 542.50, *Z* = -1.26, *p*_min_ = .29, *r* = -0.15, nor in their perceived psychological distress, *t*(68) = -0.74, *p* = .46, *d* = -0.18, 95% CI [-0.65, 0.29]. For detailed information on demographic and health data as well as absolute values see Tables 1 and 2.

### Procedure

Participants filled in the questionnaires online via LimeSurvey [52], which took about 45–60 min. Participants were reimbursed for their time with 10€ or Psychology students were offered 1 course credit.

### Measures

All questionnaires used were self-report measures in German versions. Out of a total of 12 questionnaires, only the AQ [20], the GSQ (self-developed German translation) and the SCL-90 [19] were considered in the current study.

- The *Autism-Spectrum Quotient* (AQ; [6]) is a self-report questionnaire frequently used to examine autism-like traits in individuals with and without ASD [32, 39, 50, 58, 70]. The AQ consists of 50 items, summed up to five subscales (social skills, attention switching, attention to detail, communication, imagination) with 10 items each. Individuals indicate the extent to which they agree with each statement on a 4-point Likert scale (*I strongly agree* to *I strongly disagree*). Each item is scored 1 (autism-like) or 0 (not autism-like). Total scores range between 0 to 50 points with higher scores indicating a higher expression of autism-like traits. For each participant, the AQ total score and the five AQ subscale scores were calculated. Regarding reliability, the Cronbach’s alpha of the AQ for the current sample was 0.88, so the internal consistency of the questionnaire is satisfying.
- The *Glasgow Sensory Questionnaire* (GSQ; [48]) was especially developed for adults with ASD and is appropriate for measuring sensory processing differences in individuals with and without ASD. It contains 42 items on 14 subscales concerning seven sensory modalities (visual, auditory, gustatory, olfactory, tactile, vestibular, proprioception) with six items each. Out of these six items, three each relate to the domains hypersensitivity and hyposensitivity. Individuals indicate the extent to which they experience the content of each statement on a 5-point Likert scale (0 = *Never* to 4 = *Always*). Total scores range between 0 to 168 points. The original English version has an internal consistency of *r* = 0.94 and reasonable content validity [48]. In the present study, the GSQ total score, 14 GSQ subscale scores, GSQ scores of the seven sensory modalities, and GSQ scores of the two domains hyper- and hyposensitivity were computed.
- The *Symptom-Checklist 90-R* (SCL-90-R; [16]) is an inventory for individuals aged 12 and older to assess perceived psychological distress over the past seven days. On 90 items, individuals are asked to rate how much they experience the content of each statement on a 5-point Likert scale (0 = *Not at all* to 4 = *Extremely*). Across all items, a global parameter, the Global Severity Index (GSI), can be calculated to measure the overall psychological distress. For the GSI, T-scores for students exist for interpretation (60–64 = slightly, 65–69 significantly, 70–74 strongly, 75–80 very strong increased psychological distress). The German SCL-90-R [19] was used, with an internal consistency of *r* = 0.96 for students GSI and a disputed factorial validity [19].

### Translation and cross-cultural adaptation

Following permission of the authors (Robertson & Simmons, University of Glasgow), the GSQ was translated into German. A translation-back-translation was done by two German/English bilingual psychologists followed by a discussion and revision of the translation in the research team. To enable an intercultural equivalent validation of the GSQ, the aspects of the *Universalist model of cross-cultural adaptation* [29] were considered including conceptual, item, semantic, operational, measurement, and functional equivalence.

### Data analysis

The analysis of the results followed the approach of Sapey-Triomphe et al. [51]. Where possible, we compared our data to the original English data of Robertson and Simmons [48]. However, when there were no data available as in the case of the subscale analysis, we followed Sapey-Triomphe et al. [51] by comparing our data to the data of Ward et al. [66]. In general, unlike Sapey-Triomphe et al. [51], IBM SPSS Statistics (Version 27 and Version 28) and IBM SPSS Amos (Version 27) were used for statistical analyses. One-sided tests were used with a threshold of *p* < .05 for statistical significance, and for multiple comparisons Bonferroni correction was applied. For group comparisons of correlations, the data were* z* standardized and tested two-tailed using an online Fisher’s *r*-to-*Z* Transformation [68].

To investigate the validity and compare the present scores to the scores obtained by the original English version [48] and to the scores obtained by Ward et al. [66], Spearman’s ρ and two-tailed one-sample *t* tests were performed. Regarding the mentioned comparison, it should be noted that Pearson’s *r* (used by Robertson and Simmons [48] and Sapey-Triomphe et al. [51]) and Spearman’s ρ (used in this study) are comparable because they use the same scale from -1 to 1. However, it must always be kept in mind when making this comparison that both measures are based on different assumptions [36, 53]. In our study we used Spearman’s ρ because pre-analyses showed that the premise of linearity was not given. Therefore, the use of Pearson’s *r* would have underestimated the strength of the relation between the two variables in question [17]. Internal reliability was assessed with Cronbach’s alpha and item-total correlations were considered. To test the internal structure, a confirmatory factor analysis (CFA), with unweighted least squares method (ULS), was used to verify the existing model of Robertson and Simmons [48]. In addition, to check if a better model exists, exploratory factor analyses (EFA), with principal axis factoring method (PAF) and ULS, were calculated. For rotation, varimax rotation was used, and small coefficients (absolute value below 0.30) were suppressed.

Spearman’s ρ was used for correlation analyses. Subgroups were compared using Fisher’s *r*-to-*Z* transformation [68] and two-tailed unpaired *t* tests. For within group comparisons, two-tailed paired-samples *t* tests were calculated. Between subgroups, correlation matrices for the 14 GSQ subscales were compared with Fisher’s *r*-to-*Z* comparisons [68].

## Results

### Validation process

There were no missing data. For multiple comparisons, only significances after Bonferroni correction are reported with *p*_corrected_ < .0035 for the 14 GSQ subscales, *p*_corrected_ < .007 for the seven GSQ sensory modalities, and *p*_corrected_ < .01 for the five AQ subscales.

### Validity

**Fig. 1:**
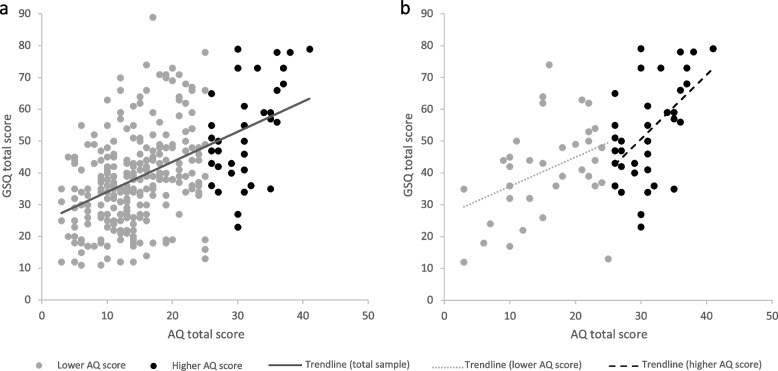
Correlations between AQ and GSQ total scores. *Note.* **a** Correlation between the AQ and GSQ total scores in the total sample in students with higher (black) and lower (grey) AQ scores, ρ = .44, *p* < .001. **b** Correlation between the AQ and GSQ total scores in the two subgroups in students with higher (black, *ρ* = .48, *p* = .002, GSQ = 0.53 × AQ + 0.07) and lower (grey, *ρ* = .38, *p* = .01, GSQ = 1.17 × AQ—0.59) AQ scores

The GSQ total scores ranged from 11 to 89 (*M* = 39.8 ± 15.4 compared to 56.7 ± 23.6 in the original English sample) and the AQ total scores ranged from 3 to 41 (*M* = 16.1 ± 7.7 compared to 22.5 ± 10.6 in the original English sample) in the total sample (see Tables 8 and 9 in Appendix 1). The GSQ and AQ total scores correlated moderately in the total sample, *ρ* = 0.44, *p* < .001 (compared to *r* = 0.78, *p* < .0001 in the original English version), see Fig. 1a.

**Fig. 2:**
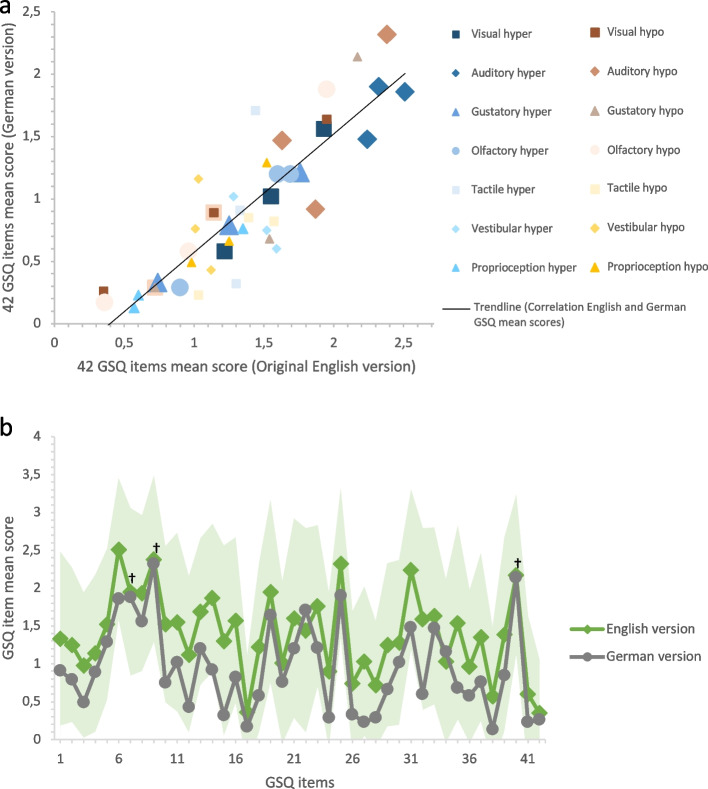
Comparison of German and original English GSQ versions. *Note.* **a** Correlations of the 42 GSQ item mean scores between the German version and the original English version [48], *ρ* = .87, *p* < .001. Items are assigned to the respective subscales with symbols (see legend). Hyper = hypersensitivity, hypo = hyposensitivity. **b** Mean scores of the 42 GSQ items in the German version (grey) and in the original English version (green; [48]). The light green area shows the area within the standard deviation for the 42 GSQ items in the original English version [48]. ^†^ *p* > .05 for Items 7, 9, and 40 in one-sample *t* test

The means and standard deviations of the 42 GSQ items of the present data correlated strongly and moderately respectively with means and standard deviations by Robertson and Simmons [48], *ρ* = 0.87, *p* < .001 (for means), and *ρ* = 0.60, *p* < .001 (for standard deviations; see Fig. 2a). As can be seen in Fig. 2b, one-sample *t* tests between means of the 42 GSQ items of the current sample and means by Robertson and Simmons [48] were not significant for Item 7, *t*(296) = -1.05, *p* = .30, *d* = -0.06, 95% CI [-0.18, 0.05], Item 9,* t*(296) = -0.99, *p* = .32, *d* = -0.06, 95% CI [-0.17, 0.06], and Item 40, *t*(296) = -0.60, *p* = .55, *d* = -0.04, 95% CI [-0.15, 0.08]. For the means of the remaining 39 GSQ items one-sample *t* tests were significant, *t*(296)_min_ > 2.01, *p*_max_ < .045, *d*_min_ > 0.12, 95% CI [0.003, 0.23].

In the total sample as well as in students with higher AQ scores, none of the 14 GSQ subscale means correlated significantly with the data by Ward et al. [66], *ρ*_max_ < 0.35, *p*_min_ > .02, after Bonferroni correction. In students with lower AQ scores, the subscale tactile hypersensitivity mean correlated with the data by Ward et al. [66], *ρ* = 0.46, *p* = .0028. Of the seven sensory modality means, none correlated with the data by Ward et al. [66] when comparing total samples as well as individuals with higher and lower AQ scores in both samples, *ρ*_max_ < 0.29, *p*_min_ > .04, after Bonferroni correction. Details on correlations are presented in Table 10 in Appendix 1.

### Reliability

The internal consistency for the German GSQ was satisfying. For the 42 items, Cronbach’s alpha was 0.88 in the total sample, 0.85 in individuals with higher AQ scores, and 0.86 in individuals with lower AQ scores. For the 14 subscales, Cronbach’s alpha was 0.87 in the total sample, 0.82 in students with higher AQ scores, and 0.85 in students with lower AQ scores. Corrected item-total correlation was 0.36 on average for the total sample, with correlations under 0.20 for Items 17, 22, and 36. It was 0.33 for students with higher AQ scores (with a negative correlation for Item 35), and 0.34 for students with lower AQ scores. Detailed item-total correlations can be found in Table 11 in Appendix 1.

### Internal structure

A visualisation of the tested model is presented in Fig. 5 in Appendix 2. The model shows the internal structure of the GSQ as presented by Robertson and Simmons [48]. All GSQ items were assigned to one of the 14 GSQ subscales. In a next step, each of these 14 GSQ subscales was assigned to one of the two domains hyper- or hyposensitivity. Our assignment corresponds to the structural construction of the questionnaire by Robertson and Simmons [48]. In order to test the model, a Standardized First-Order Confirmatory Factor Model of the GSQ’s 14 subscales was computed in a first step, followed by a Standardized Second-Order Confirmatory Factor Model of the GSQ’s two domains based on the first-order model. Twelve participants were excluded from CFA from the total sample (*N* = 297) because they were identified as outliers due to Mahalanobis distance, *p* < 10^–3^.^1^ For the remaining 285 students, the model solution for CFA in Amos was not permitted because six error variances were negative for the second-order factor model for the two domains hyper- and hyposensitivity. Therefore, we set these negative variances to 0 and re-ran the analyses. In more detail, standardized regression weights for the two domains hyper- and hyposensitivity were on average 0.88, range = 0.91. (vestibular hyposensitivity)—0.49 (olfactory hyposensitivity). For the 14 subscales, standardized regression weights were on average 0.44, range = 0.09 (Item 17, olfactory hyposensitivity)—0.72 (Item 23, gustatory hypersensitivity). The covariance between the two domains was 0.25, the correlation was 0.84. The Goodness of Fit Index (GFI) was 0.94 and the χ^2^ */df* was 0.80 (χ^2^ = 520.5, *df* = 810). Overall, the present data did not seem to fit the model presented in Fig. 5 in Appendix 2 perfectly. Then, an EFA with the 42 GSQ items in the sample of the remaining 285 students was performed to extract the underlying factor structure of the German GSQ version. The ratio of subjects per variable was 6.8. Since PAF and ULS achieved relatively similar results, only the results of the ULS are reported. The Kaiser–Meyer–Olkin measure of sampling adequacy was 0.82 (> 0.60), showing that data were appropriate for factor analysis. Bartlett’s test of Sphericity was significant (χ^2^ > 2620, *p* < .001), indicating that correlations between variables were sufficient. The determinant was 5.913E-5 (> 0.00001), so multicollinearity was not a problem. Measures of sampling adequacy (ideally > 0.60) were below 0.60 for Items 22 (tactile hypersensitivity), 17, and 36 (both olfactory hyposensitivity) and, therefore, these items should be removed from the scale. There were 29 (3%) nonredundant residuals (ideally < 60% for a good model). The average communalities across the 42 items were 0.31 before and 0.40 after rotation, with communalities under 0.30 after rotation for Items 5, 9, 17, 18, 22, 29, and 38. This means that on average the extracted factors moderately predicted participants’ responses to the items. Fourteen factors with an eigenvalue ≥ 1 could be identified which accounted for 39.5% of the total variance after rotation, which is rather low. The 14 factors were also confirmed by the scree-plot. However, as the classification of the items to the subscales differed from the one in the original English version [48], the factor loadings on the 14 factors did not reflect the original structure. Factor loadings can be found in Table 12 in Appendix 1.

Further, an ULS for two factors to be extracted was performed, only in this case with oblique rotation, to check if the two domains hyper- and hyposensitivity could be found. The Kaiser–Meyer–Olkin was 0.82 and Bartlett’s test of Sphericity was significant (χ^2^ > 2620, *p* < .001). The determinant was 5.913E-5. Measures of sampling adequacy were below 0.60 for Items 17, 22, and 36. There were 310 (36%) nonredundant residuals. Average communalities across the 42 items were 0.19 after rotation, with communalities under 0.10 after rotation for Items 5, 17, 22, 28, 36, 38, and 39 (see Table 3). This means that on average the extracted factors poorly predicted participants’ responses to the items. The total variance was 18.58%, which is quite low compared to the explained variance of 39% found by Sapey-Triomphe et al. [51], with 15.31% of variance for Factor 1 and 3.28% of variance for Factor 2 after rotation. Seventeen items loaded on Factor 1, with 13 items belonging to the domain hyposensitivity. Factor 2 included 16 negatively loaded items, 13 of them belonging to the domain hypersensitivity. Thus, the internal structure does seem to somewhat match the structure of the original English version [48] and of the French version [51], and therefore in the analyses reported below we maintain the distinction between hyper- and hypo-sensitivity to allow for comparison with these earlier studies. Individual factor loadings are shown in Table 4.

**Table 3:** Communalities for the 42 GSQ items for the two-factor solution in the total sample

	Items																				
	Item 1	Item 2	Item 3	Item 4	Item 5	Item 6	Item 7	Item 8	Item 9	Item 10	Item 11	Item 12	Item 13	Item 14	Item 15	Item 16	Item 17	Item 18	Item 19	Item 20	Item 21
Initial	.26	.29	.31	.40	.22	.42	.30	.35	.28	.34	.42	.29	.34	.39	.35	.36	.20	.26	.30	.41	.39
Extraction	.17	.15	.16	.34	.10	.32	.13	.34	.14	.29	.24	.11	.25	.27	.14	.18	.00	.19	.21	.30	.17
	Items																				
	Item 22	Item 23	Item 24	Item 25	Item 26	Item 27	Item 28	Item 29	Item 30	Item 31	Item 32	Item 33	Item 34	Item 35	Item 36	Item 37	Item 38	Item 39	Item 40	Item 41	Item 42
Initial	.15	.39	.27	.38	.27	.26	.24	.28	.36	.39	.35	.35	.35	.26	.31	.31	.22	.23	.31	.26	.30
Extraction	.02	.25	.11	.28	.14	.12	.07	.16	.21	.30	.17	.30	.36	.18	.03	.24	.09	.06	.15	.14	.26

Extraction method: unweighted least squares

**Table 4:** Pattern matrix with factor loadings of the two-factor solution in the total sample

Factors	Items																				
	Item 34	Item 42	Item 20	Item 35	Item 33	Item 4	Item 19	Item 37	Item 11	Item 26	Item 7	Item 41	Item 12	Item9	Item 16	Item 29	Item 40	Item 39	Item 5	Item 28	Item 38
Factor 1	.62	.56	.50	.50	.46	.45	.43	.42	.40	.37	.35	.34	.34	.32	.31	.31	.31	-	-	-	-
Factor 2	-	-	-	-	-	-	-	-	-	-	-	-	-	-	-	-	-	-	-	-	-
	Items																				
Factors	Item 36	Item 22	Item 8	Item 10	Item 6	Item 13	Item 25	Item 30	Item 2	Item 23	Item 1	Item 18	Item 14	Item 31	Item 21	Item 3	Item 27	Item 15	Item 24	Item 32	Item 17
Factor 1	-	-	-	-	-	-	-	-	-	-	-	-	-	-	-	-	-	-	-	-	-
Factor 2	-	-	-.60	-.59	-.54	-.52	-.49	-.45	-.44	-.44	-.40	-.39	-.39	-.38	-.38	-.37	-.34	-.31	-	-	-

Extraction method: unweighted least squares. Rotation method: direct oblimin. The rotation has converged in 10 iterations

### Sensory processing differences in students with higher and lower Autism-Spectrum Quotient scores

#### Group comparison of students with higher vs. lower Autism-Spectrum Quotient scores

Students with higher and lower AQ scores differed significantly in GSQ, *t*(68) = -3.31, *p* = .002, *d* = -0.79, 95% CI [-1.27, -0.30], and AQ total scores, *t*(58.33) = -12.12, *p* < .001, *d* = -2.90, 95% CI [-3.57, -2.22], with higher scores for students with higher AQ scores (GSQ: *M* = 53.1 ± 15.8, AQ: *M* = 31.2 ± 4.1) compared to students with lower AQ scores (GSQ: *M* = 41.0 ± 14.9, AQ: *M* = 15.7 ± 6.4).

**Fig. 3:**
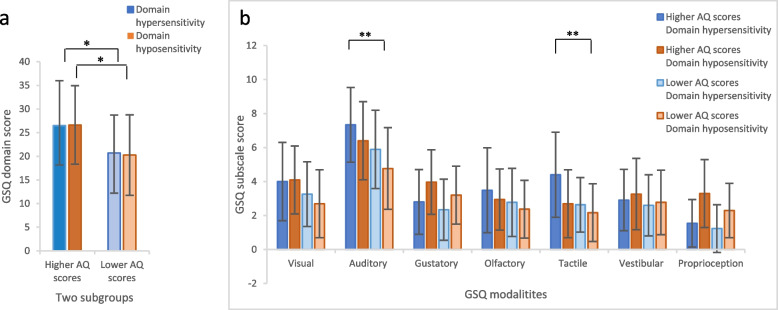
Scores of the two GSQ domains hyper- and hyposensitivity and 14 GSQ subscales in students with higher and lower AQ scores. *Note.* **a** Total scores of the two GSQ domains hypersensitivity (students with higher AQ scores: *M* = 26.5 ± 9.5, students with lower AQ scores: *M* = 20.7 ± 8.0) and hyposensitivity (students with higher AQ scores: *M* = 26.6 ± 8.3, students with lower AQ scores: *M* = 20.3 ± 8.5). **b** Total scores of the 14 GSQ subscales. Error bars result from *SD*s.* *p* < .05 for significant differences between students with higher and lower AQ scores. **significant differences between students with higher and lower AQ scores after Bonferroni correction with *p*_corrected_ < .007 for seven GSQ modalities

Regarding the two GSQ domains, individuals with higher AQ scores scored significantly higher in both domains than individuals with lower AQ scores (hypersensitivity: *t*(68) = -2.75, *p* = .008, *d* = -0.66, 95% CI [-1.14, -0.17], hyposensitivity: *t*(68) = -3.18, *p* = .002, *d* = -0.76, 95% CI [-1.24, -0.27]), see Table 9 in Appendix 1 for absolute values. Unpaired *t* tests revealed further that students with higher AQ scores scored significantly higher than students with lower AQ scores in the auditory, *t*(68) = -3.22, *p* = .002, *d* = -0.77, 95% CI [-1.25, -0.28], and tactile modality, *t*(59.69) = -3.43, *p* = .001, *d* = -0.82, 95% CI [-1.31, -0.33]. Differences regarding the remaining modalities were not significant, *t*_max_ < -2.45, *p*_min_ > .02, *d*_max_ < -0.59, 95% CI [-1.06, -0.11] (see Fig. 3).

Further, the two subgroups were compared regarding their correlation matrices. Fisher’s *r*-to-*Z* comparisons [68] between the two subgroups revealed no significant differences in correlations between the 14 GSQ subscales,* Z*_max_ < 1.71, *p*_min_ > .09, *q*_max_ < 0.43. Details on correlations between the 14 GSQ subscales are presented in Table 5.

**Table 5:**
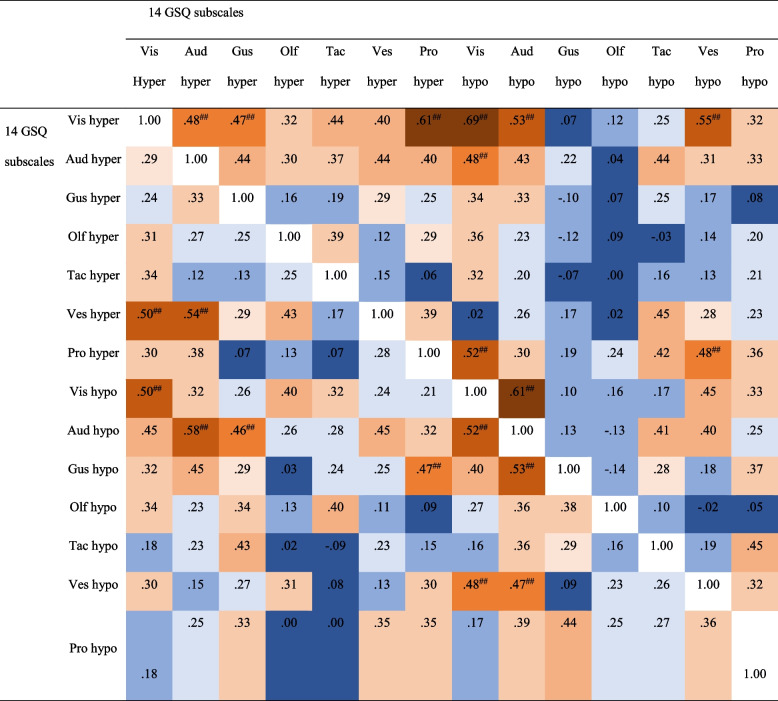
Correlation matrix with Spearman’s ρ between the 14 GSQ subscales in the two subgroups

*Hyper* hypersensitivity, *hypo* hyposensitivity, *Vis* visual, *Aud* auditory, *Gus* gustatory, *Olf* olfactory, *Tac* tactile, *Ves* vestibular, *Pro* proprioception. Correlations for students with higher AQ scores can be seen in the upper right triangle. Correlations for students with lower AQ scores are presented in the lower left triangle

^##^significant after Bonferroni correction with *p*_corrected_ < .0029 for the 14 subscales

### Correlations within subgroups

In both subgroups, GSQ and AQ total scores correlated significantly: students with higher AQ scores: *ρ* = 0.48, *p* = .002, students with lower AQ scores: *ρ* = 0.38, *p* = .01 (see Fig. 1b). A Fisher’s *r*-to-*Z* comparison [68] between the two subgroups revealed no significant difference in these two correlations,* Z* = 0.49, *p* = .62, *q* = 0.12.

In individuals with higher AQ scores, the GSQ total score and none of the AQ.subscales correlated significantly. In individuals with lower AQ scores, the GSQ total score and two AQ subscales correlated significantly. Details on correlations are presented in Table 6. Further, in students with higher and lower AQ scores, only the hypersensitivity domain correlated significantly with the AQ total score. Regarding the modalities, in individuals with higher AQ scores, there were significant correlations for two modalities with the AQ total score and in individuals with lower AQ scores for one modality with the AQ total score. Detailed information on correlations can be found in Table 6. Overall, correlations were weak to moderate. Individuals with higher and lower AQ scores did not differ significantly in their correlations (see Table 6).

**Table 6:** Correlations (Spearman’s ρ) between GSQ and AQ

	Students with higher AQ scores		Students with lower AQ scores		Students with higher vs. lower AQ scores		
Correlations between	*ρ*	*p*	*ρ*	*p*	*z* Test Statistic	*p*	Cohen’s *q*
GSQ total score and …							
AQ total score	.48*	.002	.38*	.01	0.49	.63	0.12
Five AQ subscales							
AQ social interaction	.18	.15	-.06	.36	1.00	.32	0.25
AQ attention switching	.34	.02	.42^#^	.006	0.38	.70	-0.10
AQ attention to detail	.40	.02	.40^#^	.009	0.00	1.00	0.00
AQ communication	.17	.16	.33	.03	0.67	.50	-0.17
AQ imagination	.13	.22	.28	.05	0.62	.54	-0.16
AQ total score and …							
Two GSQ domains							
GSQ hypersensitivity	.55*	< .001	.36*	.02	1.00	.32	0.25
GSQ hyposensitivity	.28	.05	.29	.05	0.04	.97	-0.01
Seven GSQ modalities							
GSQ visual	.60^##^	< .001	.13	.23	2.22	.03	0.56
GSQ auditory	.27	.06	.56^##^	< .001	1.45	.15	-0.36
GSQ gustatory	.25	.07	.37	.01	0.53	.60	-0.13
GSQ olfactory	.17	.17	.20	.12	0.13	.90	-0.03
GSQ tactile	.55^##^	< .001	.12	.24	1.95	.051	0.49
GSQ vestibular	.27	.06	.20	.13	0.33	.74	0.08
GSQ proprioception	.38	.01	.06	.37	1.36	.17	0.34

In the right column two-tailed significances for Fisher’s *r*-to-*Z* transformation are noted, with a *z* critical value from -1.96 to 1.96

^*^ *p* < .05. ^#^significant after Bonferroni correction with *p*_corrected_ < .01 for five AQ subscales. ^##^significant after Bonferroni correction with *p*_corrected_ < .007 for seven GSQ modalities

**Fig. 4:**
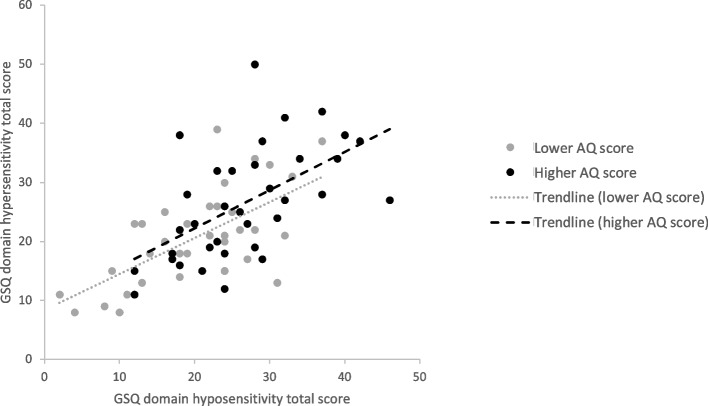
Correlation of GSQ domain hyper- and hyposensitivity domain scores in students with higher and lower AQ scores. *Note.* Students with higher AQ scores (black): *ρ* = .63, *p* < .001. Students with lower AQ scores (grey): *ρ* = .57, *p* < .001

Within subgroups, hyper- and hyposensitivity domain total scores were significantly positively correlated, with *ρ* = 0.63, *p* < .001 for students with higher AQ scores and *ρ* = 0.57, *p* < .001 for students with lower AQ scores (see Fig. 4). A Fisher’s *r*-to-*Z* comparison [68] between the two subgroups revealed no significant difference in these two correlations,* Z* = 0.38, *p* = .71, *q* = 0.09. Regarding the seven sensory modalities, only for the visual and the auditory modalities the hyper- and hyposensitivity scores correlated significantly for students with higher and lower AQ scores. The two subgroups did not differ in their correlations between hyper- and hyposensitivity scores. Details on correlations between the two domains for each sensory modality and on comparisons between subgroups regarding these correlations are presented in Table 7.

**Table 7:** Correlations (Spearman’s ρ) between the two GSQ domains hyper- and hyposensitivity

	Students with higher AQ scores		Students with lower AQ scores		Students with higher vs. lower AQ scores		
Correlations between the hypersensitivity and hyposensitivity scores within each modality	*ρ*	*p*	*ρ*	*p*	*Z* Test Statistic	*p*	Cohen’s *q*
Domains in total	.63*	< .001	.57*	< .001	0.36	.72	0.09
Visual modality	.69^##^	< .001	.50^##^	.001	1.22	.22	0.31
Auditory modality	.43^##^	.005	.58^##^	< .001	0.80	.42	-0.20
Gustatory modality	-.10	.29	.29	.05	1.59	.11	-0.40
Olfactory modality	.09	.30	.13	.22	0.17	.87	-0.04
Tactile modality	.16	.18	-.09	.30	1.01	.31	0.25
Vestibular modality	.28	.05	.13	.22	0.63	.53	0.16
Proprioception modality	.36	.02	.35	.02	0.05	.96	0.01

In the right column two-tailed significances for Fisher’s *r*-to-*Z* transformation are noted, with a *z* critical value from -1.96 to 1.96

^*^ *p* < .05. ^##^significant after Bonferroni correction with *p*_corrected_ < .007 for seven GSQ modalities

Hyper- and hyposensitivity domain scores did not differ significantly within subgroups, *t*_max_ < 0.39, *p*_min_ > .70, *d*_max_ < 0.07, 95% CI [-0.27, 0.40]. Regarding the seven modalities, both subgroups had significantly higher hyposensitivity scores in the proprioception modality (higher AQ scores: *t*(34) = -5.26, *p* < .001, *d* = -0.89, 95% CI [-1.28, -0.49], lower AQ scores: *t*(34) = -3.85, *p* < .001, *d* = -0.65, 95% CI [-1.01, -0.28]). Further, students with lower AQ scores had significantly higher hypersensitivity scores in the auditory modality, *t*(34) = 3.04, *p* = .004, *d* = 0.51, 95% CI [0.16, 0.86]. Students with higher AQ scores showed significantly higher hypersensitivity scores in the tactile modality, *t*(34) = 3.39, *p* = .002, *d* = 0.57, 95% CI [0.21, 0.93]. There were no other significant differences between hyper- and hyposensitivity scores, *t*_max_ < -2.57, *p*_min_ > .015, *d*_max_ < -0.44, 95% CI [-0.78, -0.09], after Bonferroni correction. See Table 9 in Appendix 1 for absolute values.

## Discussion

The current study aimed at (a) validating the German version of the GSQ in students with higher and lower AQ scores according to the Universalist model of cross-cultural adaptation [29] and (b) at replicating the sensory processing differences of individuals with higher or lower AQ scores as reported in the original English version by Robertson and Simmons [48] and in the French version of Sapey-Triomphe et al. [51]. In the present study, with the present sample, the validation of the German GSQ was not completely satisfying. Also, the sensory processing differences could not be replicated. In the following, the results will be discussed.

### Validation of the German Glasgow Sensory Questionnaire

#### Validity

Like expected, GSQ and AQ total scores correlated moderately, with ρ = 0.44, in the present study, suggesting that autism-like traits go along with sensory sensitivity. Horder et al. [31] report a similar correlation of *r* = 0.48. However, this correlation was higher in the original English version, *r* = 0.78 [48], as well as in other validation studies, *r* = 0.81 [51], *r* = 0.75 [35], *r* = 0.63 [59]. There were also studies reporting even lower correlations of *r* = 0.23 [67], and *r* = 0.25 [63]. Ward et al. [67] state that differences might possibly be explained by ethnic differences in samples, as studies with higher correlations mostly came from Western countries (English, French, Danish validation studies), while studies with lower correlations came from Eastern countries (Chinese, Japanese validation studies). This, however, does not explain the lower correlation in the present Western sample. Instead, the present moderate correlation could be explained by the fact that in the present sample only very few individuals had AQ total scores ≥ 26 (*n* = 35). Robertson and Simmons [48] had 54 individuals with AQ scores ≥ 28 (thereof 39 individuals with AQ scores ≥ 32). Sapey-Triomphe et al. [51] even had 102 participants with ≥ 26 points on the AQ, including some individuals with an ASD diagnosis. Also against expectations, the present total sample had a lower mean GSQ total score than the sample in the original English version by Robertson and Simmons [48]. Probably, the present scores in the GSQ were lower because of the lower AQ scores, as more autism-like traits go along with higher sensory sensitivity [48, 51]. The standard deviations between the German and the original English version [48] also correlated moderately. The mean scores of each GSQ item correlated strongly between these two GSQ versions even though the scores were of lower magnitude overall in the present sample. Furthermore, the correlation coefficients regarding mean scores and standard deviations of each GSQ item were in line with the ones obtained by Sapey-Triomphe et al. [51]. The strong correlation between the German version and the English version could be explained by the fact that the items were well translated into German and therefore have a similar meaning in the two versions. The mean scores in the present sample were consistently lower than in the English sample, which could be attributed to the sample’s nature. This might also be responsible for the non-significant correlations with data by Ward et al. [66], including individuals with an ASD diagnosis. Furthermore, Ward et al. [66] included beneath individuals with an ASD diagnosis also synesthetes. Overall, the validity was not completely satisfying.

### Reliability

Like expected and reported in other validation studies [35, 48, 51, 59, 63, 66], the German GSQ showed a good internal consistency. Against expectations, corrected item-total correlations were only acceptable on average, and lower than the ones reported in Sapey-Triomphe et al. [51]. In students with higher AQ scores, Item 35 even had a negative item-total correlation. Possibly, this item is only weakly related to the other items, or it was seen as very ambiguous by the present sample. Interestingly, not only in the present study but also in other studies, correlations for Items 17, 22, and 36 were very low [51, 63]. This indicates that these items might also be ambiguous or not sufficiently informative. So, caution may be needed for their interpretation.

### Internal structure

Finally, against expectations, with EFA, the original 14 subscale structure could not be found. It was, however, possible to approximately recreate the two domains with an EFA, but not with the CFA. Problematic items again seemed to be numbers 17, 22, and 36. Accordingly, Items 17 and 36 also had strikingly low communalities in analyses by Sapey-Triomphe et al. [51]. Both items assess olfactory hyposensitivity. Item 22 assesses tactile hypersensitivity. These items seem to be problematic in general. Items with low communalities could be removed from the questionnaire or they could indicate an additional factor [13]. Both approaches do not seem appropriate in the present case. On the one hand, removing items would result in the subscales consisting of only one or two items. A scale should, however, consist of three items at least [42]. On the other hand, additional factors would lead to too many subscales. Another approach could be to renew the problematic items so that they capture the respective sensory sensitivity more accurately.

### Sensory processing differences in students with higher and lower Autism-Spectrum Quotient scores

#### Group comparison of students with higher vs. lower Autism-Spectrum Quotient scores

Like expected and in line with Robertson and Simmons [48] and Sapey-Triomphe et al. [51], the mean scores of GSQ and AQ differed significantly between students with higher and lower AQ scores, with higher scores for individuals with higher AQ scores. Further, all GSQ subscore means of individuals with higher AQ scores were higher than those of individuals with lower AQ scores in the present sample. Not in all cases, however, these differences were statistically significant. This could be attributed to the fact that the current sample obtained too few individuals with very high AQ scores. Thus, the subgroups did not differ strongly enough.

Students with higher AQ scores did not show stronger correlations between the 14 subscales than students with lower AQ scores. This is in contrast to expectations and to Sapey-Triomphe et al. [51], who found stronger correlations for individuals with higher AQ scores. Also in contrast to predictions and Sapey-Triomphe et al. [51], the correlation matrices for the hypersensitivity domain did not differ between the subgroups. However, in line with expectations and with Sapey-Triomphe et al. [51], the correlation matrices for the hyposensitivity domain did not differ between the two subgroups. Thus, it was not possible to illustrate that individuals with higher and lower AQ scores differ in their sensory processing differences. Possibly, the GSQ cannot provide information about differing sensory processing differences between individuals with higher and lower AQ scores when the surveyed subgroups are not sufficiently different which was probably the case in the current study.

### Correlations within subgroups

Like expected, the correlations between AQ and GSQ total scores were positive in the two subgroups. In contrast to expectations, neither the GSQ total score was positively associated to all the five AQ subscales nor the AQ total score was positively related to all GSQ domains and modalities in both subgroups. The nature of the sample could also be responsible for this result.

Further, in line with expectations and Sapey-Triomphe et al. [51], the scores for the hyper- and hyposensitivity domains were positively related in both subgroups in the present study. This suggests that experiencing hypersensitivity goes along with also experiencing hyposensitivity, indicating sensory processing differences (hyper- and hyposensitivity across modalities). Current correlations were only a bit lower than the ones in Sapey-Triomphe et al. [51]. However, contrary to expectations, in the present sample, the hypersensitivity domain score was not significantly higher than the hyposensitivity domain score in both groups. Consequently, participants seemed to experience hyposensitivity to the same degree as hypersensitivity. This is also in line with studies that report that individuals with ASD are affected by hyper- as well as hyposensitivity [7, 28, 40]. Regarding correlations and expressions of the hyper- and hyposensitivity domains for all seven sensory modalities, the expected consistent patterns (higher GSQ scores for individuals with higher than lower AQ scores, higher hypersensitivity than hyposensitivity scores) could not be found. Again, the sample’s nature could be responsible for this result. Especially for the result that individuals with higher AQ scores did not have higher GSQ scores than individuals with lower AQ scores. However, the fact that hyper- and hyposensitivity scores did not differ could also be attributed to the fact that individuals perceive both types of sensory sensitivity in the same way. This would fit with study results that assume that individuals with ASD are not mainly affected by hypersensitivity, but also by hyposensitivity [7, 28, 40].

### Strengths and limitations

A strength of the current study is the recruitment of a large student sample. The sample was widely and evenly distributed across different fields of study, including both genders and individuals with mental health issues. There were no missing data. However, in the current study, the long German AQ version was used, despite Freitag et al. [20] advising to use the short version *AQ-k* due to the insufficient discriminatory power of some items of the long version. Nevertheless, in the present study, the long version was used in order to stay as close as possible to other validation studies of the GSQ and enable a comparison of the results. In addition, an exploratory analysis using the shorter AQ version showed a similar pattern of results. Future studies should, however, consider the use of the shorter AQ version in German. This also has the advantage that it takes less time to fill in. Another limitation concerns the sample of the present study. Whereas, Sapey-Triomphe et al. [51] included 245 individuals (114 females, *M*_age_ = 32.1, with 13.7 years in formal education on average) in their study, 95 of which reported having a formal ASD diagnosis and Robertson and Simmons [48] assessed data of 212 individuals (142 females, *M*_age_ = 26.75) including two individuals with a formal ASD diagnosis, we recruited a sample of students without individuals with an ASD diagnosis. One could argue that this is not very representative of the general population. However, Robertson and Simmons [48] also included students and colleagues in their study and only 2 of their participants reported having a formal ASD diagnosis. They still demonstrated the investigation of sensory sensitivity using the GSQ using AQ score ranges to define their groups. Unfortunately, unlike Robertson and Simmons [48] the AQ scores in our sample were not as evenly distributed.

Contrary to expectations, only 35 out of 297 students had AQ scores higher than 26 in our sample. Therefore, the present sample seems not suitable for drawing meaningful conclusions about the sensory processing differences of individuals with higher AQ scores. The present results seem more consistent with the sensory processing differences found for individuals with lower AQ scores as reported in Sapey-Triomphe et al. [51]. It seems that the GSQ is less informative for the general population if there are not enough individuals with higher AQ scores in the sample. Further investigations should therefore not only include more individuals with higher AQ scores, but particularly individuals with an ASD diagnosis. This would be appropriate as the questionnaire was developed especially for people on the spectrum [48]. It could also lead to more satisfying results and a better basis for comparisons.

### Implications and perspectives

As presented above, individuals with ASD can be affected negatively by their sensory sensations [2, 21, 22, 48, 57]. However, sometimes their sensory sensitivity goes along with rather positive consequences [48, 57]. In either case, it is important to have a good insight in which way sensitivity prevails to support them as good as possible in their respective situation (e.g., reducing stress by creating rooms with low noise levels if it is known that an individual reacts to sounds with sensory hypersensitivity, or empowering individuals who have very sensitive sense of smell and could use that as a professional strength for example as a perfumer). For this purpose, due to its multifaced structure, the GSQ is an appropriate questionnaire. However, in order to use the GSQ reliably and trustworthily a satisfactory validation should be provided. This was not the case in this study for all considered validation aspects. Further efforts should, thus, be made to validate the German GSQ successfully including more individuals with higher AQ scores. Furthermore, Items 17, 22, and 36 should be considered for a revision.

## Conclusions

In the current study, the AQ and GSQ scores were lower than in some other validation studies. Validity was moderate to (very) low for the investigated aspects. The German GSQ showed a good internal consistency, but only acceptable corrected item-total correlations. Still, the internal structure found within the present sample differed from the original English version’s structure [48]. So, conceptual, item, semantic and functional equivalence remain questionable. The replication of the sensory processing differences was not successful which can possibly be attributed to the nature of the sample. Further investigations with more individuals with higher AQ scores should be performed to ensure a valid use of the GSQ in German language in individuals with ASD. If in these studies the discussed items are also conspicuous, consideration should be given to replacing the respective items.

## Figures and Tables

**Figure:**
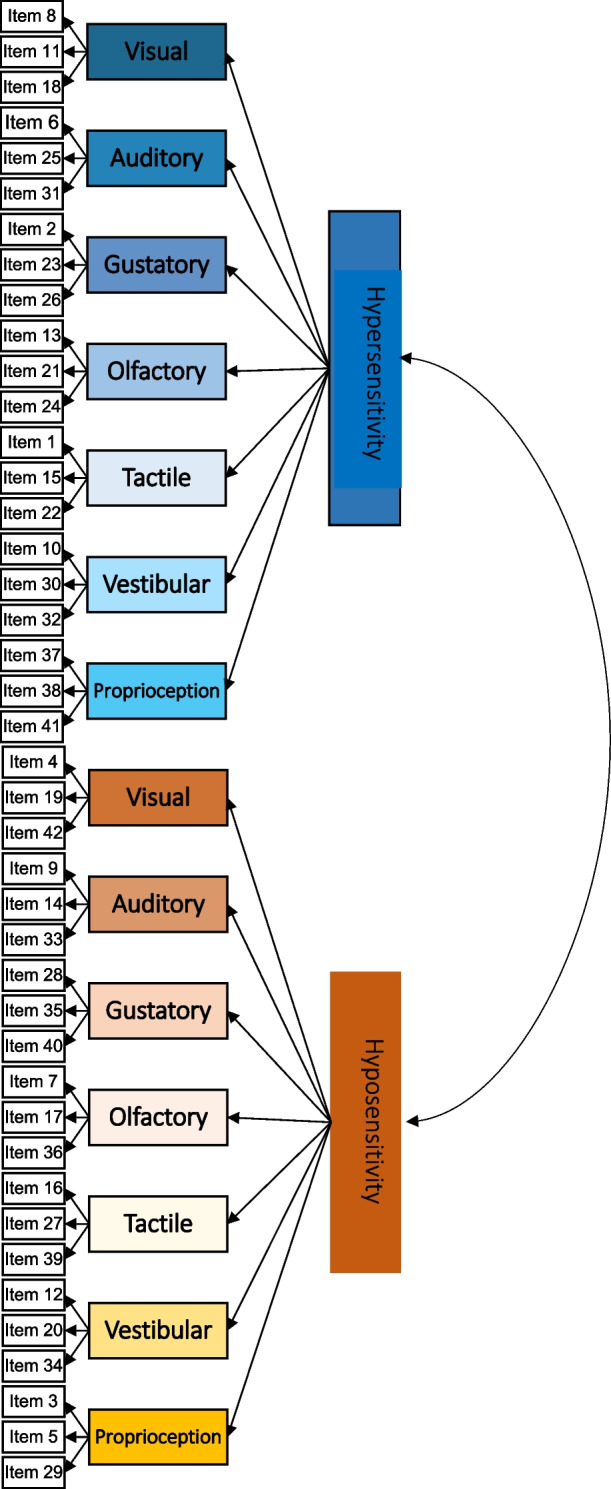
**Fig. 5** Internal structure of the Glasgow Sensory Questionnaire (GSQ). *Note.* Tested second-order model. All GSQ items assigned to one of the 14 subscales. Each subscale assigned to one of the two domains

## Data Availability

The datasets used and/or analysed during the current study are available from the corresponding author upon reasonable request for purposes such as replication and/ or meta-analyses. The German version of the GSQ is freely available from the corresponding author (Melanie.Ring@uniklinikum-dresden.de). Regarding the English version of the GSQ, please contact David Simmons (David.Simmons@glasgow.ac.uk) and/ or Ashley Robertson (Ashley.Robertson@glasgow.ac.uk).

## Appendix: Additional detailed information on statistical procedures and results

### Appendix 1: Further tables

**Table:** **Table 8** Descriptive statistics for AQ

	All subjects (*N* = 297)	Students with higher AQ scores (*n* = 35)	Students with lower AQ scores (*n* = 35)
Descriptive statistics AQ	*M (*± *SD)*	*M (*± *SD)*	*M (*± *SD)*
AQ total score	16.1 (± 7.7)	31.2 (± 4.1)	15.7 (± 6.4)
AQ subscale social interaction	2.3 (± 2.4)	6.5 (± 1.9)	2.3 (± 2.2)
AQ subscale attention switching	4.1 (± 2.4)	7.5 (± 1.5)	4.4 (± 2.4)
AQ subscale attention to detail	4.6 (± 2.3)	6.9 (± 2.0)	4.4 (± 2.3)
AQ subscale communication	2.0 (± 1.9)	5.3 (± 1.8)	1.8 (± 1.8)
AQ subscale imagination	3.0 (± 2.0)	5.1 (± 1.5)	2.8 (± 1.7)

**Table:** **Table 9** Descriptive statistics for GSQ

	All subjects (*N* = 297)	Students with higher AQ scores (*n* = 35)	Students with lower AQ scores (*n* = 35)
Descriptive statistics GSQ	*M (*± *SD)*	*M (*± *SD)*	*M (*± *SD)*
GSQ total score	39.8 (± 15.4)	53.1 (± 15.8)	41.0 (± 14.9)
GSQ domains			
GSQ domain hypersensitivity	19.9 (± 8.6)	26.5 (± 9.5)	20.7 (± 8.0)
GSQ domain hyposensitivity	20.0 (± 8.3)	26.6 (± 8.3)	20.3 (± 8.5)
GSQ modalities			
GSQ modality visual	6.0 (± 3.3)	8.1 (± 4.0)	5.9 (± 3.4)
GSQ modality auditory	10.0 (± 3.9)	13.7 (± 3.8)	10.7 (± 4.2)
GSQ modality gustatory	5.4 (± 2.6)	6.8 (± 2.6)	5.5 (± 2.8)
GSQ modality olfactory	5.3 (± 2.7)	6.4 (± 3.3)	5.1 (± 2.7)
GSQ modality tactile	4.8 (± 2.7)	7.1 (± 3.3)	4.8 (± 2.2)
GSQ modality vestibular	4.7 (± 2.9)	6.2 (± 3.1)	5.4 (± 2.7)
GSQ modality proprioception	3.6 (± 2.4)	4.8 (± 2.8)	3.5 (± 2.5)
GSQ subscales			
GSQ subscale visual hypersensitivity	3.2 (± 1.9)	4.0 (± 2.3)	3.3 (± 1.9)
GSQ subscale auditory hypersensitivity	5.3 (± 2.3)	7.3 (± 2.2)	5.9 (± 2.3)
GSQ subscale gustatory hypersensitivity	2.3 (± 1.7)	2.8 (± 1.9)	2. 3(± 1.8)
GSQ subscale olfactory hypersensitivity	2.7 (± 2.0)	3.5 (± 2.5)	2.8 (± 2.0)
GSQ subscale tactile hypersensitivity	2.9 (± 1.8)	4.4 (± 2.5)	2.6 (± 1.6)
GSQ subscale vestibular hypersensitivity	2.4 (± 1.6)	2.9 (± 1.8)	2.6 (± 1.8)
GSQ subscale proprioception hypersensitivity	1.1 (± 1.4)	1.5 (± 1.4)	1.2 (± 1.4)
GSQ subscale visual hyposensitivity	2.8 (± 1.9)	4.1 (± 2.0)	2.7 (± 2.0)
GSQ subscale auditory hyposensitivity	4.7 (± 2.2)	6.4 (± 2.3)	4.8 (± 2.4)
GSQ subscale gustatory hyposensitivity	3.1 (± 1.7)	4.0 (± 1.9)	3.2 (± 1.7)
GSQ subscale olfactory hyposensitivity	2.6 (± 1.8)	2.9 (± 1.8)	2.4 (± 1.7)
GSQ subscale tactile hyposensitivity	1.9 (± 1.6)	2.7 (± 2.0)	2.2 (± 1.7)
GSQ subscale vestibular hyposensitivity	2.4 (± 2.0)	3.3 (± 2.1)	2.8 (± 1.9)
GSQ subscale proprioception hyposensitivity	2.4 (± 1.6)	3.3 (± 2.0)	2.3 (± 1.6)

**Table:** **Table 10** Correlations (Spearman’s ρ) between German version and data by Ward et al. [66]

	All subjects			Individuals with higher AQ scores	Individuals with lower AQ scores	
GSQ subscores	ρ	*p*	ρ	*p*	ρ	*p*
Fourteen GSQ Subscales						
Visual hypersensitivity	-.04	.29	.16	.18	.14	.21
Auditory hypersensitivity	-.07	.20	.09	.30	-.08	.33
Gustatory hypersensitivity	-.11	.08	-.06	.36	-.23	.09
Olfactory hypersensitivity	.03	.34	.16	.18	-.01	.48
Tactile hypersensitivity	.11	.08	.23	.09	.46^##^	.0028
Vestibular hypersensitivity	-.07	.18	-.09	.31	-.03	.44
Proprioception hypersensitivity	.01	.47	.16	.18	.23	.10
Visual hyposensitivity	-.07	.18	.26	.07	.07	.35
Auditory hyposensitivity	-.09	.13	.35	.02	.22	.10
Gustatory hyposensitivity	-.08	.16	.07	.35	.28	.05
Olfactory hyposensitivity	.13	.06	.003	.49	.13	.22
Tactile hyposensitivity	-.12	.07	-.13	.23	-.07	.35
Vestibular hyposensitivity	-.02	.40	-.08	.33	.13	.23
Proprioception hyposensitivity	-.05	.27	-.04	.42	-.09	.30
Seven GSQ modalities						
Visual	-.03	.38	.25	.08	.19	.14
Auditory	-.09	.12	.29	.04	.18	.15
Gustatory	-.10	.11	.02	.46	.06	.36
Olfactory	.10	.11	.16	.18	-.05	.39
Tactile	-.07	.20	.001	.50	.19	.14
Vestibular	-.02	.40	-.12	.24	.12	.24
Proprioception	.03	.33	.04	.41	.06	.36

^##^significant after Bonferroni correction with *p*_corrected_ < .0035 for 14 GSQ subscales. There were no significant correlations after Bonferroni correction with *p*_corrected_ < .007 for seven GSQ modalities

**Table:** **Table 11** Corrected item-total correlation of the 42 GSQ items in the three groups

Groups	Items																				
	Item 1	Item 2	Item 3	Item 4	Item 5	Item 6	Item 7	Item 8	Item 9	Item 10	Item 11	Item 12	Item 13	Item 14	Item 15	Item 16	Item 17	Item 18	Item 19	Item 20	Item 21
Total sample	.39	.28	.30	.53	.26	.49	.33	.51	.38	.33	.43	.23	.39	.48	.36	.38	.07	.35	.42	.53	.34
Higher AQ scores	.41	.24	.32	.54	.41	.57	.07	.63	.42	.18	.44	.30	.32	.49	.39	.55	.09	.42	.43	.39	.26
Lower AQ scores	.06	.44	.43	.44	.20	.60	.26	.52	.52	.49	.32	.19	.44	.54	.27	.27	.16	.48	.44	.37	.22
Groups	Items																				
	Item 22	Item 23	Item 24	Item 25	Item 26	Item 27	Item 28	Item 29	Item 30	Item 31	Item 32	Item 33	Item 34	Item 35	Item 36	Item 37	Item 38	Item 39	Item 40	Item 41	Item 42
Total sample	.11	.49	.34	.50	.30	.29	.24	.37	.39	.53	.39	.54	.49	.26	.13	.44	.36	.23	.36	.36	.39
Higher AQ scores	.04	.50	.07	.59	.33	.41	.20	.23	.29	.49	.33	.36	.37	-.03	.03	.51	.24	.10	.29	.25	.37
Lower AQ scores	.13	.49	.01	.38	.28	.27	.34	.38	.42	.32	.29	.54	.33	.46	.41	.24	.21	.14	.34	.40	.35

**Table:** **Table 12** Rotated factor matrix with factor loadings of the 14-factor solution in the total sample

Items	Factor loadings													
	Factor 1	Factor 2	Factor 3	Factor 4	Factor 5	Factor 6	Factor 7	Factor 8	Factor 9	Factor 10	Factor 11	Factor 12	Factor 13	Factor 14
Item 6	.66	-	-	-	-	-	-	-	-	-	-	-	-	-
Item 25	.58	-	-	-	-	-	-	-	-	-	-	-	-	-
Item 8	.48	-	-	-	-	-	-	-	-	-	-	-	-	-
Item 10	.38	-	-	-	-	-	-	-	-	-	-	-	-	-
Item 13	.34	-	-	-	-	-	-	-	-	-	-	-	-	-
Item 1	.33	-	-	-	-	-	-	-	-	-	-	-	-	-
Item 18	.32	-	-	-	-	-	-	-	-	-	-	-	-	-
Item 30	-	-	-	-	-	-	-	-	-	-	-	-	-	-
Item 34	-	.53	-	-	-	-	-	-	-	-	-	-	-	-
Item 4	.30	.50	-	-	-	-	-	-	-	-	-	-	-	-
Item 33	-	.48	-	-	-	-	-	-	-	-	-	-	-	-
Item 35	-	.44	-	-	-	-	-	-	-	-	-	-	-	-
Item 37	-	.43	-	-	-	-	-	-	-	-	-	-	-	-
Item 42	-	.42	-	-	.31	-	-	-	-	-	-	-	-	-
Item 9	-	.39	-	-	-	-	-	-	-	-	-	-	-	-
Item 15	-	-	.69	-	-	-	-	-	-	-	-	-	-	-
Item 20	-	.36	.37	-	-	-	-	-	-	-	-	-	-	-
Item 3	-	-	.37	-	-	-	-	-	-	-	-	-	-	-
Item 2	-	-	-	.65	-	-	-	-	-	-	-	-	-	-
Item 23	-	-	-	.51	-	-	-	-	-	-	-	-	-	-
Items	Factor 1	Factor 2	Factor 3	Factor 4	Factor 5	Factor 6	Factor 7	Factor 8	Factor 9	Factor 10	Factor 11	Factor 12	Factor 13	Factor 14
Item 11	-	-	-	-	.59	-	-	-	-	-	-	-	-	-
Item 12	-	-	-	-	.55	-	-	-	-	-	-	-	-	-
Item 21	-	-	-	-	-	.72	-	-	-	-	-	-	-	-
Item 24	-	-	-	-	-	.45	-	-	-	-	-	-	-	-
Item 39	-	-	-	-	-	-	.50	-	-	-	-	-	-	-
Item 28	-	-	-	-	-	-	.49	-	-	-	-	-	-	-
Item 29	-	-	-	-	-	-	.36	-	-	-	-	-	-	-
Item 16	-	-	-	-	-	-	.35	-	-	-	-	-	-	-
Item 32	-	-	-	-	-	-	-	.51	-	-	-	-	-	-
Item 38	-	-	-	-	-	-	-	.40	-	-	-	-	-	-
Item 19	-	.33	-	-	-	-	-	.36	-	-	-	-	-	-
Item 41	-	-	-	-	-	-	-	-	.50	-	-	-	-	-
Item 31	-	-	-	-	-	-	-	-	.43	-	-	-	-	-
Item 40	-	-	-	-	-	-	-	-	-	-	-	-	-	-
Item 36	-	-	-	-	-	-	-	-	-	.54	-	-	-	-
Item 5	-	-	-	-	-	-	-	-	-	.41	-	-	-	-
Item 26	-	-	-	-	-	-	-	-	-	-	.52	-	-	-
Item 7	-	-	-	-	-	-	-	-	-	-	.37	-	-	-
Item 27	-	-	-	-	-	-	-	-	-	-	-	.47	-	-
Item 14	-	-	-	-	-	-	-	-	-	-	-	-	-	-
Item 17	-	-	-	-	-	-	-	-	-	-	-	-	.48	-
Item 22	-	-	-	-	-	-	-	-	-	-	-	-	-	.43

Extraction method: unweighted least squares. Rotation method: varimax. The rotation has converged in 25 iterations

## Abbreviations

ASD: Autism Spectrum Disorder
AQ: Autism-Spectrum Quotient
CFA: Confirmatory Factor Analysis
EFA: Exploratory Factor Analyses
GSI: Global Severity Index
GSQ: Glasgow Sensory Questionnaire
PAF: Principal Axis Factoring
SCL-90: Symptom-Checklist
TUD: Technische Universität Dresden
UKD: Universitätsklinikum Carl Gustav Carus Dresden
ULS: Unweighted Least Squares

## Footnotes

1 These outliers were, however, not identified in any of the other analyses and were, therefore, not excluded from other analyses presented in this paper.
